# Office Design’s Impact on Psychosocial Work Environment and Emotional Health

**DOI:** 10.3390/ijerph21040438

**Published:** 2024-04-03

**Authors:** Christina Bodin Danielsson, Töres Theorell

**Affiliations:** 1 Department of Architecture and Civil Engineering, Chalmers University of Technology, 412 96 Gothenburg, Sweden; 2 The Royal Institute of Technology (KTH), School of Architecture and Built Environment, 100 44 Stockholm, Sweden; 3 Department of Psychology, Stress Research Institute, Stockholm University, 106 91 Stockholm, Sweden; tores.theorell@su.se; 4 Department of Global Health, Karolinska Institutet, 171 77 Stockholm, Sweden

**Keywords:** office design, office work environment, psychosocial work environment, social health, emotional health, emotional exhaustion, Job Demand–Control–Support model, gender

## Abstract

This study explores the association between office design and (a) the psychosocial work environment and (b) the emotional health among 4352 employees in seven different office designs. A multivariate linear regression analysis was performed with adjustments for age and educational level for men and women separately. Results show that psychosocial factors and emotional exhaustion differ between both office designs and between genders, with best outcomes in cell offices, except for psychological demands that are rated the most favourable in shared-room offices. Cell offices and small open-plan offices show a strong beneficial association with emotional exhaustion in women. Among men, hot-desking is most problematic regarding psychosocial work environment and emotional exhaustion. Women rate the psychosocial environment low in combi-office and report emotional exhaustion in small open offices.

## 1. Introduction

A healthy organization can be defined as an organization characterized by both financial success, i.e., profitability, and a physically and psychologically healthy workforce [1] (p. 462). The recognition of this builds on the insight that the work environment consists of three dimensions—the physical, the psychosocial, and the organizational. 

Today, the office is the most common workplace in the western world [2]. This combined with the fact that the workplace is a central environment in many people’s daily lives—since 40% of a fulltime employee´s waking hours are spent working—highlights the importance of the office workplace. Not only do people spend a lot of time at the office, it is also a centre for social interaction and for building social networks [3]. The workplace importance to people’s social life is reflected in the fact that poor work environments are costly at both individual and societal levels in terms of personal suffering and costs for society [4,5]. Psychosocial work environment is the concept that refers to how the individual experiences and responds to his or her surroundings at work [6]. Accordingly, the impact of the psychosocial work environment for employee health and well-being is well recognized in the field of occupational health [7] and more recently also at focus in organizational psychology [8,9]. Accordingly, the psychosocial work environment is well researched in relation to occupational health, including the office employee cohort. The physical environment´s role has been less researched, especially its relation to psychosocial factors and its potential emotional health consequences. Its role for employee environmental satisfaction or performance has instead gained more interest [10,11]. Given the psychosocial work environment´s established impact on employee health [7], the lack of research on potential associations between psychosocial and physical environments is remarkable, especially that of offices. The office workplace holds not only a dominant role in contemporary working life, but it is also central in the social life and well-being of many employees. All combined, this calls for a greater attention to the physical environment´s role from the occupational health and organizational psychology perspective, especially regarding the office environment. There was a call for further scientific exploration of this in a previous article on employee satisfaction with workspace contribution in different office designs [12]. However, when approaching this unexplored field of occupational health and organizational psychology, an exploratory approach is necessary since the use of such an approach reduces the risks of overlooking potentially important factors. When addressing issues of importance to the field it is important to be concrete and to introduce possible practical implications. HR practitioners mention this as a reason why academic research may have failed to have an impact on workforce performance and well-being. It becomes inadequate as a source of guidance [13]. 

## 2. Theoretical Background

### 2.1. The Psychosocial Components of the Office Workplace

Research tells us that the psychosocial work environment is critical for employee well-being. For example, low skill discretion, besides potentially giving rise to low accomplishment, may be associated with emotional exhaustion, mental problems, and depersonalization [14,15]. High psychological demands, combined with low decision-making possibilities, are associated with psychological distress [16]. Good skill discretion on the other hand as well as good decision authority in the workplace improve psychological functioning, self-rated health, and coping style [17]. In addition, good decision authority predicts job satisfaction [18]. These factors are associated with high commitment, which decreases employee turnover [19]. 

Interpersonal relationships are central to well-being, and this is true also at work, i.e., towards colleagues and supervisors. Good social support from colleagues has several positive effects [14], e.g., reduced risks for future depression and anxiety disorders [20]. Meanwhile low social support combined with high psychological demands further increases the risk of depression and anxiety among employees [12,20,21]. Workplace conflicts could have negative health outcomes, e.g., increased levels of strain [22], rates of work disability [23], and poor self-rated health and fatigue [24,25]. Relationships to supervisors are central for employee well-being [26]. These influence subordinates´ health, stress levels, and sickness absence [27]. When emotional health problems in modern society as a consequence of increased complexity are discussed, a central factor is the work environment, e.g., [28].

### 2.2. Job Demand–Control–Support Model 

A frequently utilized theoretical model in the study of the environmental part of the psychosocial job situation is the JDCS (Job Demand–Control–Support) model. The job demand–control–support model for characterizing psychosocial work characteristics was originally proposed by Karasek [29]. It was constructed with the purpose of estimating the degree of adverse/beneficial psychosocial components in work design. The construction of the model was a reaction against a previous view that confined all the measurements of the effects of psychosocial factors at work to the individual´s own perception and characteristics. Therefore, all the questions in the questionnaires used in demand–control–support research are formulated in order to capture the demands the individual is exposed to, the possibility the environment gives the employee to exert control, and finally the amount of support the environment provides. Although the model was constructed during a period when piece work provided the model for work environment analysis, it has turned out to be a robust model that can be applied to many different kinds of work environments and is still used extensively (see for instance [30]).

The model has three main components: Psychological demands, Decision Latitude, and Social Support. According to the model, high psychological demands, low decision latitude (a combination of decision authority and skill discretion), and poor support all have adverse health effects [29,31,32].

All three components have subdimensions. With regard to psychological demands there are both quantitative (how much) and qualitative (how difficult) aspects. Decision latitude (“control”) has two distinct aspects: skill discretion, which is the possibility the environment gives the employee to develop skills for exerting control—for instance, in unexpected situations; and decision authority, which is the amount of everyday democracy regarding work tasks that the environment provides. Support could be summarized as the amount of instrumental and emotional support that the employee is provided by the environment. The questionnaire that is used in the present study is an abbreviated form of the original one. Its psychometric properties have been found to be satisfactory [33], and furthermore, the analyses indicated that is it statistically appropriate to separate the two control dimensions into its two components: skill discretion and decision authority. 

In the following text, we sometimes refer to the JDC model (Job Demand—Control), which is the same model without the support dimension. A relatively recent Cochrane review [19] based upon 25 prospective studies showed that the scientific level of relationship between low decision latitude and poor support at work on one hand and emotional exhaustion on the other hand reached the level of moderately strong evidence (level 3 of 4). High psychological demands only reached the level of limited evidence (level 2 of 4) in relation to emotional exhaustion in that study. In this study, we focus on all three components of JDC model demands, decision latitude and social support, with decision latitude divided into skill discretion and decision authority as separate dimensions. All of these could be assumed to interact with the physical office environment in generating health effects. Our focus is on the potential impact of the office environment on employees´ perception of psychosocial factors here. 

### 2.3. The Physical Components of the Office Workplace 

Despite the fact that the office is the workplace for many people today [2], there are large knowledge gaps regarding the office design´s association to employees’ health and well-being. The focus in occupational health research on the office environment has been on environmental factors and design characteristics as well as the health outcomes directly related to those, e.g., impact of noise, privacy, and density/crowding. The major research gap with regard to health has to do with the office environment´s relation to psychosocial environment and specific psychosocial factors and how this in turn is associated with employee emotional health. If any relationships exist, how do those relate to different office designs? The discussion on these relationships has been influenced by the experiences during the COVID-19 pandemic. 

Existing research shows that open offices are overall less beneficial than others [34]. This is especially the case for traditional open plan offices, which have the most negative influence on employee general health [35] and sickness absence [36], but also on noise and privacy [37]. The hypothesis is that these problems can be ascribed to exposure to environmental stressors, particularly since there is a lack of control over the environmental situation. This finds support in a more recent study on office design´s contribution to job satisfaction, to pleasantness, and to job effectiveness [12]. It showed that satisfaction with one´s own contribution as an employee relates to the access to supportive facilities in the office, enabling control of the noise and privacy situation, potentially improving the psychosocial climate, as noise per se negatively may increase workplace conflicts [38]. Job satisfaction research has shown that talk privacy has a significant relationship with job satisfaction [39], something reflected in lower job satisfaction in open plan offices. Thus, indications exist that office design influences the psychosocial work environment. But we do not know what potential impact different office designs may have on the psychosocial environment.

Although most studies focus on the negative effects of open office plans, there are some positive aspects of sharing workspace, including psychosocial benefits. For example, proximity between colleagues is important for several reasons, including support in social networks that decreases with physical distance [40]. Proximity, besides social support, promotes knowledge transfer in the office by facilitating interaction and friendship development, which is in turn beneficial to psychosocial work environment and health [41]. Sharing workspace also benefits employee–manager relationships, as merely seeing and hearing managers within the workspace make employees perceive them as more friendly [42]. Thus, in medium-sized open plan offices where employees and managers often share workspace, the leadership is also rated more positively [43]. Further, workplace conflicts, according to another study by Bodin Danielsson and colleagues [38], seem to be less frequent in offices with ten or more people. 

In summary, the importance of a good psychosocial work environment for employees’ well-being is well established. But the role of the physical environment including the office environment has been less explored. The present study on office design’s impact on the psychosocial workplace climate is accordingly intended to fill a gap [12]. 

### 2.4. Gender 

There are documented gender differences in both working conditions and health outcomes that include worse health outcomes (including stress, sick leave rates) among women than men, partly attributed to gender differences, e.g., regarding risk factors and responsibilities [44,45]. Other differences of potential relevance in an office context have also been shown regarding work relations and communication patterns, e.g., that men are more work-focused in their conversation, while women are more relation-oriented [20,46,47]. Regarding the physical aspects of the office, indications of differences of potential relevance to office design are reported, e.g., more sensitivity to environmental stimuli among women than men in shopping environments in terms of receptiveness to information and detail [48], and to noise disturbances [38,49,50]. Among women with high job complexity, noise problems correlate with absenteeism [51]. Additionally, the office type´s impact on sick leave is stronger for female employees [36]. Women also report less satisfaction than men with office design’s beneficial impact on the work environment [12]. The same study also showed more dissatisfaction among men in small open plan offices, potentially attributed to a greater sensitivity to crowding among men [52]. Regarding workplace conflicts, traditional open plan offices appear to be more negative for men [38], while for women the combi-office is problematic. This may be due to its high degree of teamwork, an in-built conflict risk [53]. Women may exhibit more sensitivity due to the heightened importance of work relationships for their well-being [20]. 

## 3. Purpose 

This exploratory study is motivated by the psychosocial work environment´s importance for employee wellbeing and the office design´s potential impact on this. The fact is that most research on office environment on employee well-being investigates it with an ambient factor perspective, i.e., how privacy and noise situation influence employee well-being. The rare research that investigates health in relation to office environment does this on a general level, without incorporating the psychosocial perspective that is central for employee health. To our knowledge, the potential association between impact of the physical work environment on the psychosocial has not been investigated before. Based on this, the present study is a response to the call for further knowledge on the matter [12]. We do this by using the four psychosocial factors—psychological demands, social support, decision authority, and skill discretion that the original JDC (Job Demand–Control—with control constituted by the combination of decision authority and skill discretion) model is based on (see Section 2.2. above). Although it is well established that these psychosocial factors influence employees’ health status, we do not know how this relates to the physical workplace, which, for the majority in the western world, is an office [2]. This lack of knowledge concerns both how these four psychosocial factors may be associated individually and jointly with office employees’ health. According to our knowledge, the potential association of these factors to office design has previously not been studied. 

Outcomes that are associated with the psychosocial work environment are job satisfaction, comfort, and performance, as well as access to supportive facilities. It applies the same study design and data set as the initial study. That the present study represents a step further in this work is further emphasized by the fact that the initial study had a more “architectural” approach in that it examined the extent to which employees perceive the office design to be satisfactory for its purpose. The present study on the other hand illuminates how these office designs are perceived with regard to psychosocial work environment and extent of emotional exhaustion.

**Figure 1:**
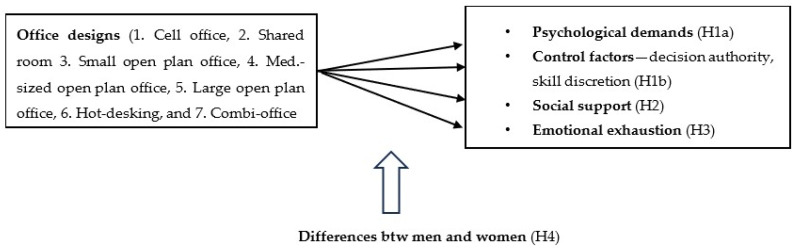
Conceptual Model of Study Design. *Note:* Statistical analysis is based on conceptual model.

All the psychosocial factors of the JDCS model are taken into account. Besides the psychosocial factors, we also investigate the office design’s influence on emotional exhaustion in employees (for conceptualization, see Figure 1). We recognize that outcomes like performance and employee turnover are also relevant but are not included in this study. 

These are our working hypotheses:

**H1a.**  *Psychological demands are perceived as higher among employees in traditional open plan offices than in other office designs due to greater exposure to environmental stress.*

**H1b.**  *In contrast to demands, both control factors (decision authority, skill discretion) are reported as favourable among employees in cell offices, i.e., individual rooms, compared to employees in other office designs.*

The possibility in cell offices to control exposure to environmental stressors will then, according to the JDCS model, decrease stress.

**H2.**  *Good social support is more prevalent in office designs with open plan layout, where workspaces and facilities are shared, rather than in cell offices.*

The resource of social support from the colleague network at the workplace, according to the JCDS model, will then be promoted by the interaction between colleagues that is facilitated by physical proximity between people.

**H3.**  *Employees report less emotional exhaustion in office designs where they describe the psychosocial factors positively.*

According to the JDCS model, positive psychosocial factors are beneficial for employee emotional health. Hence, if employees report psychosocial factors in a certain office design, their emotional health should also be better.

**H4.**  *Finally, we hypothesize that there are differences between men and women (H1–H3)*.

Given the documented gender differences in both working conditions and health outcomes in occupational health research, found also in JDCS model-based research, the matter is worth further investigation. 

## 4. Methods 

Methodologically, our study is exploratory, as it investigates the office environment´s association to psychosocial factors, something previously not studied. As a follow up of a previous exploratory study, it applies a tentative approach that can be described as exploratory in two steps.

### 4.1. Sample and Questionnaire 

Using the same sample, we follow up on the 2018 study on employees’ satisfaction with workspace contribution in different office designs [12]. It is based upon the 2012 wave of the Swedish Longitudinal Occupational Survey of Health (SLOSH)—a nationally representative longitudinal cohort study of work environment and health, including organizational aspects and physical work environment, e.g., office types. SLOSH also covers background data about participants. All combined, this makes SLOSH useful for the purpose of our study. Conducted biennially, data are collected by paper-and-pencil questionnaires [54,55], with an internet questionnaire option offered in 2012. SLOSH is a unique prospective study on work environment and health, providing a basis for improvements of work environments in Sweden and other countries. SLOSH is conducted at the Stress Research Institute at Stockholm University, Sweden. (For details: https://www.su.se/english/research/research-projects/slosh (accessed on 8 February 2024)). 

From the SLOSH 2012 wave, 18,915 participated in our study. From these, an analytic sample was extracted of 4352 participants (54.7% women, 45.3% men), which remained after exclusion of participants who lacked information on crucial items for our study (see Table 1). For the individual items used, the analytic sample varied, as information was missing from some of the crucial items for our study. Exact numbers on each item are presented in each specific table (i.e., Table 2, Table 3 and Table 4). Participants not working in an office, or who did not fill in information regarding office design, resulted in a significant sample loss (>1000 participants).

#### 4.1.1. Descriptive Analysis of Background Factors in the Study Sample

Our descriptive results regarding background factors (age, education, job rank, supervisory position) show significantly different distributions across the office designs for both genders (see both Table 1 above and Table A1 in Appendix A). The gender distribution also differed significantly between the office designs studied. 

The sample consisted of more women (54.7%) than men, reflected in all office designs. The largest proportion of participants were cell-office employees, (n = 2084, w = 46%, m = 50%). The smallest proportion worked in combi-offices, (n = 81, 2% of both genders). For individual background factors, we found, with regard to gender, that next after cell offices the highest proportion of both genders worked in shared-room offices (w = 19%, m = 18%), and the lowest proportion in combi-offices, for both genders. The greatest difference in gender distribution was found in hot-desking offices (w = 9%, m = 6% of total sample). The highest mean age was found in cell offices (w = 51.5 yrs, m = 52.5 yrs). The youngest mean age was found for women in large open plan (w = 46 yrs) and for men in medium-sized open offices (m = 47.5 yrs).

With regard to educational level, both men and women in cell offices held the largest proportion of employees with low education (w = 42%, m = 54%), but also of high education (w = 49%, m = 51%), while a larger proportion of women than men with low education worked in the three traditional open plan offices. Despite a higher mean educational level among women, men had higher job ranks (SEI). The highest proportion of women with high job ranks worked in cell offices (39.4%), while for men this was in both combi-offices (48.5%) and large open-plan offices (47%). The highest proportion of managers with staff responsibility was found in cell offices (w = 19%, m = 33%). A smaller proportion in supervisory positions was found among women in traditional open plan offices (medium-sized, 77%, and small open plan offices, 75.5%) and among men in small open plan offices (64%) and hot-desking offices (63%). 

### 4.2. Office Definitions and Outcome Variables 

#### 4.2.1. Independent Variable—Office Designs

The participants in our study worked in one of seven office designs (see Table 5). There are seven office types established in contemporary office architecture as defined by the combination of architectural and functional features functional features (for definitions and illustrations of office types, see, e.g., [37,56]). The architectural features are physical, of which the spatial organization (i.e., plan layout) is the most prominent, while the functional features relate to the actual work taking place and the organization of this (i.e., functions related to the use of the office). Features are determined by factors such as functional needs and technical feasibility, e.g., ICT (Information Communication Technology). It also works in the opposite direction, i.e., technical and functional possibilities lead to new organizations of work affecting the architectural design. The established office types are prototypes, hence “cross-pollinations” between defined office types exist. For example, there exist traditional open offices that incorporate positive characteristics of the activity-based office types, where employees have good access to back-up rooms for meetings, private conversations, etc. that typical traditional open offices lack. The present exploratory study uses the same seven office designs as in the previous study [12], of which this is a follow up study. The office designs are: (1) cell office (individual room), (2) shared-room office (2–3 people/workspace), (3) small open-plan office (4–9 people in workspace), (4) medium-sized open-plan office (10–24 people/workspace), (5) large open-plan office (≥25 people/workspace), (6) combi-office (team-based activity-based office type), and (7) hot-desking office (a subcategory to activity-based office type flex-office, A-FO). (For office designs used, see Table 5).

Our initial sample analyses revealed that participants with non-personal workstations did not work in the activity-based office-type flex-office (A-FO) as expected, but in its sub-category hot-desking office. More specifically, those with non-personal workstations reported no good access to supportive facilities like spaces for concentrated or collaborative work, defining architecture features of the A-FO. Nor was high decision authority reported, which is also a functional feature of A-FO. Due to the absence of these defining features, the term office design instead of office type is used in this article. The classification of the participants into the seven office designs was carried out by the participants themselves, since a unique combination of specific questions in the SLOSH questionnaire gives rise to the classification of office types/office designs. Questions cover ownership of workstations, spatial organization of individuals sharing the workspace combined with job characteristics, and access to supportive workspaces. A combination of the two latter dimensions gave rise to the label hot-desking offices. For information regarding SLOSH, see reference [57].

#### 4.2.2. Dependent Variable—Psychosocial Factors

Focusing on office design’s influence on employees’ perception of the psychosocial environment, our main category of dependent outcome variables were the four psychosocial factors in the JDC model: (1) *psychological demands,* (2) *decision authority,* (3) *skill discretion, and* (4) *social support.*

Questions used to assess the psychosocial environment originate in the Swedish short form (DCQ) of the Job Content Questionnaire [29,58,59], which has proved to hold good psychometric properties [33]. The questions used are:

(1) Psychological demands were assessed by five questions. Example of question asked: *“Are there often conflicting demands of your work?”*
(2) Decision authority was assessed by two questions. Example of question asked: *“Do you have the freedom to decide how your work should be performed?”*
(3) Skill discretion was assessed by four questions. Example of question asked: *“Does your work require skills?”*
(4) Social support was assessed by six statements. Example of statement: *“It is a good cohesion at my workplace.”*

The 4-point Likert response scale for the five items for psychological demands and the two control items decision authority and skill discretion ranged from “Yes, often” (1) to (4) “Almost never/Never.” However, the 4-point response scale for six items for social support ranged from “Fully agree” (1) to (4) “Completely disagree”. The sum scores for the four psychosocial factors were as follows: (1) For psychological demand, the five sub–items gave a sum score range between 5 and 20. High scores mean more psychological demands, with scores reversed for question number four; (2) for decision authority, the sum scores for the two sub-items gave a score range between 2 and 8; (3) for skill discretion, the four sub-items gave a score range between 4 and 16. High scores correspond to more control, with scores reversed for question number four for skill discretion; (4) for social support, the six sub-items gave a score range between 4 and 24. High scores mean a good outcome, i.e., high level of social support. 

#### 4.2.3. Dependent Variable—Emotional Health

Office designs’ influence on employees’ health and well-being was assessed by our second category of dependent outcome variable: (5) Emotional exhaustion. It was measured using six items (corresponding to the subdimension emotional exhaustion) from Maslach Burnout Inventory [60], which measures feelings, specifying how often the participants feel in accordance with five statements. Example of statement: *“I feel emotionally drained by my work.”*

The 6-point Likert response scale went from “every day” (1) to “a few times a year or less/Never” (6). The sum of the five items formed a summary index resulting in score sums between 5 and 30 with a reversed scale; a high score corresponds to a bad outcome, i.e., high degree of emotional exhaustion. 

### 4.3. Statistical Analyses

Investigating the association between office design and employees’ perception of the psychosocial environment as well as their emotional health, our main analysis used multivariate linear regression models. In line with the purpose of this exploratory study, the independent variable office design was used as the main explanatory variable in our analysis. In the analysis, we had two dependent variable categories: (1) psychosocial work environment, assessed by the four psychosocial factors of the Job Demand Control Support Model, and (2) emotional health, measured by the employees’ self-reported emotional exhaustion. In the analysis, we adjusted for the background factors age and education that were treated as covariates. We limited, based on statistical rationales, our selection to these two covariates. Age has an established influence on health and satisfaction with the work situation, potentially influencing the psychosocial work environment at the office. The other covariate education is important per se but was also chosen because of its relation to job rank, supervisory position, and socioeconomic status. For classification of occupations, SLOSH uses the Swedish Standard Classification of Occupations 2012 (SSYK 2012) [61], based on the International Classification of Occupations 2008 (ISCO-08) [62], for classifying and aggregating data about occupations in administrative registers or statistical surveys. However, only education was used as a covariate in the multivariate statistics since collinearity precludes concomitant use of the others as covariates. (For distribution of job rank and supervisory position in sample, see Table A1 in Appendix A).

Linear regression was chosen because it provides maximal retention of statistical variance and because the scales for both the four psychosocial factors and emotional exhaustion were close to normally distributed. The independent variable, office design, used for previously described reasons, included six out of the seven identified office types and one subcategory of one office type, with cell office as the reference category in our analyses. The rationale behind this is that cell offices clearly differ from the other office designs, which all in varying degrees reflect the sharing of workspace and amenities between colleagues.

The results of our analyses are presented as means with 95% confidence intervals for men and women separately. A higher number for a given variable compared with cell office for a specific office design represents a higher likelihood, and a lower value vice versa. Accordingly, a higher value for a given outcome variable for psychological demands and emotional exhaustion means more psychological demands and emotional exhaustion, i.e., a worse situation in comparison with cell office, while a lower value indicates a better situation. On the other hand, a higher value for decision authority and skill discretion means more control for the individual employee, i.e., a better situation in comparison with cell office, the opposite applies for lower value. 

The computations were performed by JMP, version 11.2. For the multivariate analyses, the program first tests the total effect of our explanatory variable, that is, office design, as well as the independent effects of age and education. Comparisons between distributions of men and women were made in a few cases, but the main strategy with regard to gender was that separate gender analyses were performed. Secondly, there are post hoc tests for each office design after adjustment for age and education. Demographics presented in Table 1 include gender, mean age, and education (low/middle/high). We also analysed job rank (SEI, Swedish Socio-economic classification) and supervisory position (see Table A1 in Appendix A). 

## 5. Results

### 5.1. Office Design’s Associations with Psychosocial Work Environment

The analyses of the psychosocial work environment, assessed by the four psychosocial factors, is presented in Table 2 and Table 3. There was a statistically significant influence of gender (both genders combined) on all psychosocial factors, except for psychological demand (*p*-values available at request), which was reported to be on the same level among men and women. All the other background factors (office design “in general”, age, and education) had a significant influence on psychological demands, with the exception of office design, which had no influence on psychological demands among men. For social support the case was the opposite, i.e., only office design “in general” had a significant influence and only on men (see Table 2). Regarding control at work (decision authority and skill discretion), we found for decision authority that all background factors had significant influence except for education level in women. For skill discretion, age alone showed an influence in women. For men, both office design and education proved to be significant (see Table 3). 

#### 5.1.1. Psychological Demands and Social Support (Table 2)

For *psychological demands*, in the gender separate analysis adjusted for age and education, we found for both men and women a positive influence (lower perceived psychological demands) of one office design—the shared room. In the comparisons between office types, we noted significantly lower psychological demands for men in the cell office (*p* = 0.046) and women (*p* < 0.0001) than in other office designs. Additionally, the combi-office had among women a significantly negative association to psychological demands (*p* < 0.019). For *social support*, the analysis showed a significant association to office design among men, but not among women. Accordingly, there was significantly less social support among men working in small open-plan offices (*p* < 0.008) and in hot-desking offices (*p* < 0.036). Among women, we found only tendencies, but no significant influence of individual office designs. 

#### 5.1.2. Control—Decision Authority and Skill Discretion (Table 3)

For *decision authority*, the gender separate analysis showed among both men and women negative associations to specific individual office designs. There were fewer but stronger cases of a negative influence of office designs on men, e.g., in both shared-room offices (m = *p* < 0.009, w = *p* < 0.037) and hot-desking offices (m = *p* < 0.0003, w = *p* < 0.002). Among women, two additional cases of negative influence on office design were found in small open-plan offices (*p* < 0.0007) and in combi-offices (*p* < 0.026), indicating an overall greater negative influence of office designs on *decision authority* for women than for men. With regard to *skill discretion*, we found the only statistically significant association to office design among women, with negative associations in large open plan (*p* < 0.0009) and combi-offices (*p* < 0.012).

### 5.2. Office Design’s Association with Emotional Exhaustion

The analyses of emotional exhaustion are presented in Table 4. In terms of influence of background factors per se on emotional exhaustion, analyses showed that gender had a statistically significant influence. The other background factors (age, education, and office design “in general”), which were analysed for men and women separately, showed a significant influence of all the three factors for men; for women, all factors except education had an influence. 

#### 5.2.1. Emotional Health (Table 4)

For emotional exhaustion, the analysis showed differences in influence of the various office designs on employees’ emotional exhaustion, but also between men and women. Among men, only one significant negative association between emotional exhaustion and office design was found—for hot-desking offices (*p* < 0.007). Among women, three significant associations were found. For the small open plan office and the combi-office (*p* < 0.009 resp. *p* < 0.046), a negative influence in comparison to the cell office was found, and for large open plan offices, to the contrary, a positive influence, i.e., less emotional exhaustion than in cell offices (*p* < 0.038), was found. 

## 6. Discussion

In the discussion, we refer mainly to Graphic Table 6 that gives a graphic overview of our findings. 

Our exploratory results reveal differences in the statistical impact of various office designs on various aspects of the psychosocial work environment and of emotional exhaustion, as well as between men and women. Foremost, a pattern in the impact of the office designs is revealed (see Graphic Table 6), which may be a consequence of how well the defining features of the office designs (see Table 5) either hinder or support the adverse/beneficial effects of the psychosocial factors of the JDCS model on employee emotional exhaustion. In the following section, this is exemplified. Besides these findings, an important contribution of our exploratory study is its expansion of the field of occupational health into an area previously not researched within the field—the physical work environment´s relation to the psychosocial work environment. Another contribution concerns the method we apply. Using the psychosocial factors of the classic JDCS model, we link the field of occupational research to the field of environment psychology and architecture with potential theoretical and practical contributions to both fields and their different approaches to the work environment. Despite the two fields´ interconnection in the workplace, their interplay has been under-researched particularly with regard to the association of the physical work environment to various aspects of the psychosocial work environment related to job satisfaction, workplace conflicts, and stress levels [35,38,63]. How the results of previous studies potentially relate to different psychosocial factors that make up the psychosocial work environment has been explored in our study. This implies a potential to contribute to a theoretical development of the field of occupational research. Likewise, our exploratory results have the potential to contribute to the theoretical development of the two fields of environmental psychology and of architecture, towards the field of occupational health. Regarding education, this may also have implications in both architecture and occupational health education, as a recognition of the fact that psychosocial aspects of office design may require a need to take this into account more systematically. In addition to this, our results enable practical contributions to both perspectives on work environment by offering access to other perspectives. Yet another practical contribution of our study results can be tested in real office settings by professionals that work hands-on with work environment strategies and workplace designs (e.g., HR, facility managers, architects, and interior designers).

### 6.1. Correspondence between Working Hypotheses and Results

Although this is an exploratory study, i.e., not hypothesis testing, we formulated five working hypotheses as a provisional basis for further occupational research on the office environment´s role for a sustainable working life. The correspondence between our working hypotheses and our results were the following:

Working Hypothesis 1a, according to which higher psychological demands among employees in traditional open plan offices was expected (due to a higher level of exposure to environmental stressors), was not fully supported. We did, however, for both genders find statistical tendencies of a negative impact resulting in high psychological demands in these office designs. Two office designs had a statistically significant impact. High demands were reported in the combi-office only among women. Lower demands were found for both genders in the shared room office (two to three people/room). Regarding our working hypothesis 1b, according to which cell office employees were expected to report a higher level of control (decision authority, skill discretion), this was overall supported—although to various degrees in comparison to other office designs. For decision authority, the finding was significantly lower scores among employees in shared-room and hot-desking offices, and for women also in small open plan offices and combi-offices.

Working Hypothesis 2, according to which more social support would be reported among employees in office designs with open plan layouts, i.e., shared workspaces and facilities, in comparison to cell offices, was not supported. The two cases of significant impact of office design were for small open-plan offices and in hot-desking offices, they were negative and only among men. For cell offices, only a tendency toward less social support was found among women.

Working Hypothesis 3, which predicted a lower prevalence of emotional exhaustion, i.e., better emotional health, in office designs where a good psychosocial work environment is reported, was only partially supported. However, in the opposite extreme, in small open offices and in hot-desk offices, the highest prevalence of emotional exhaustion was found—with a clear gender difference.

Our final working Hypothesis 4 on gender differences was that there would be such differences throughout previous Hypotheses 1–3. This was based on other research on differences in social interplay and environmental sensitivity between men and women. As expected, we found that individual office designs had more impact on women than men. For women, there was a larger number of significant observations with regard to impact of office designs and fewer examples of positive impact—with the exception of hot-desking offices, which had more impact on men. 

### 6.2. A Pattern of Certain Office Designs Standing Out

Summarizing our results in Graphical Table 6, a pattern emerges that we present in the graphical overview where certain office designs stand out by significant associations with our outcomes. Most of them were negative. Foremost, our exploratory results show that in comparison to the cell office, our reference, very few office designs were beneficial. 

Why, then, do certain office designs exert such an influence on the psychosocial work environment and increased risk of emotional exhaustion among employees? Based on the JDCS model connecting the psychosocial factors to the physical features, this study seeks the answers to how well different office designs facilitate the handling of strain caused by demands in the work environment. In turn, determined by how these designs support or hinder employees’ personal control, the designs affect the employees´ risk of developing emotional exhaustion. In line with this, we believe that the negative and few positive psychosocial outcomes are effects of their characteristics, which we here exemplify in different office designs. 

In the hot-desking office, the low access to supportive facilities together with non-personal workstations increase exposure to unwanted visual and acoustic stimuli. These features add to employee strain as they reduce the possibility for the employee to exert personal control, which is central to employee health according to the JDCS model. Regarding emotional exhaustion, we find a significantly negative impact of this office design that also correlates with a significantly negative impact on both high psychological demand and low decision authority. In addition, the small open-plan office lacks supportive facilities, as most work is expected to be performed at the personal workstations. Why this traditional open plan office is more negative than the other two is unclear. It may be a consequence of the smaller group size (four to nine people/room) as background speech is less disturbing than speech with high intelligibility [64]. The risks of both high speech intelligibility and low availability of privacy in small open-plan offices are greater than in larger ones. Also, work is not always collaborative. That may explain why in our study we do not find a high level of social support in open-plan offices, which we had expected. Again, negative outcomes with regard to these two psychosocial factors correlate with a higher level of emotional exhaustion among women in this office design. In contrast, we find employees in shared-room offices reporting more social support and also reporting lower levels of psychological demands. These positive psychosocial effects may explain the high level of job satisfaction in this office design found in other research [35]. The office designs that stand out by their impact on psychosocial environment and employees’ emotional exhaustion in our study are approximately the same designs in which employees report good satisfaction with the workspace´s contribution [12]. 

Some gender differences stand out in our findings. These concern both an overall stronger impact on women and that office design per se has a greater impact on women (see Graphic Table 6). The pronounced overall impact on women was particularly evident in large open-plan offices and combi-offices. Despite a greater impact among women, the strongest significant impact of an individual office design was identified among men—in the hot-desking office. If we allow some speculation, the gender differences in impact may be attributed to a greater sensitivity to environmental stimuli among women, as was initially suggested. On the other hand a more negative impact of crowding has been observed in men in the same spatial conditions [52].

Our analysis showed only one office design, the shared-room office (two to three people/room), to have equally positive and negative influence on both genders. Also, it is only in this office design that we find some support for our working hypothesis that sharing workplaces is good for the psychosocial work environment. This is possibly attributed to the office design’s small group size and proximity to colleagues, a key for friendships to develop, e.g., [41]. That this was only among women may be due to greater focus on and importance of work relations for their well-being [65]. The reason why we find significant impact of the combi-office only on women may be the same one, as the combi-office is defined by a great degree of team work with in-built conflict risks, e.g., [53]. Again, satisfaction with workspace contribution is found to be lower in this office design [12].

### 6.3. Methodological Considerations

The major strengths of this study, based on a large and national survey like SLOSH, is its representative sample of office workers. Another strength of a large occupational health survey like SLOSH is its extensive information about work environment and health. There were also limitations, however. One possible limitation relates to its exploratory nature. Being an exploratory study, it is rather hypothesis generating and not hypothesis testing in the traditional sense, although in a tentative manner we explore five working hypotheses. This is a natural consequence of the fact that this is the first step in investigating an issue previously not studied. Furthermore, another limitation is the use of self-reported data like in most cross-sectional surveys. Thus, a risk of common method bias exists in our study [66], although this problem should not be overestimated [67]. There is, e.g., a limited number of items that define office types in SLOSH 2012. For example, it covers information on employees’ office types, like number of people sharing workspaces, access to supportive facilities (e.g., spaces for concentrated work, meeting rooms etc.), but lacks details (distances to or exact numbers). It also covers functional features, like work characteristics (e.g., collaboration and decision authority) (see Table 5 for office definitions). Information on collaboration and decision authority revealed that participants reporting not having a personal workstation also did not work in flex-offices, as expected, but in its sub-group hot-desking. Lack of detailed information regarding environmental factors may lead to misclassification of a number of employees with regard to office type/office designs. This is likely, however, to result in an underestimation of associations due to random error. There is no reason to assume systematic bias in the classification. Nevertheless, observations of the office environments would have been beneficial, although such observations are very difficult to conduct in a national survey like SLOSH. 

Regarding choice of covariates, two general factors of potential importance, age and education, have been taken into account in the confounding analyses. Type of job is of course important in the study of relationships between office type and psychosocial factors. However, type of job is intrinsically involved in office type, so it is impossible to disentangle them in these kinds of analyses. In modern office development, there is a general trend towards homogeneity, so if anything, type of work as such may presently be of decreasing importance in the studied relationships. Further, a limitation with a large national survey is that it is impossible to know whether the participants chose to work in their specific office designs or not. We have insufficient knowledge about non-participants. Presumably, the office design is in many cases not an active choice by the employee but comes with the employment. Hence, the personal environment fit will then vary between individuals, affecting the psychosocial outcomes. The central idea in the Person Environment Fit model is that the knowledge and characteristics of the employee should match the characteristics of the job [68]. Finally, another limitation caused by SLOSH’s wide coverage of research subjects is the lack of subject focus that may cause confusion about the purpose of the office environment questions. This could explain why: (a) critical questions about office design were sometimes not filled in by participants, and (b) the responses were not always consistent. This could contribute to inconsistencies in the exposure measurement and an underestimation or exaggeration of the effect of office design in our study. 

## 7. Conclusions

Our exploratory results indicate that some office designs are not only associated with the psychosocial work environment, but also with the degree of emotional exhaustion. This influence reveals a pattern in which some office designs may have a stronger effect than others—mainly negative effects. We suggest that this pattern of differences can be ascribed to the defining architectural and functional features of these office designs that either support or worsen psychosocial conditions. This may explain part of the variations in employees’ degree of emotional exhaustion. However, our results are exploratory. They constitute a response to a previous call for research on the relation of office design to the psychosocial work environment [12]. There is a need for further studies. More longitudinal studies are needed since such studies may allow more causal inferences. The need for more knowledge about the office environment’s role for the psychosocial workplace climate has been accentuated during the experiences from the COVID-19 pandemic. It has thrown light on the importance of the physical workplace for employee well-being as a motivation and cohesion factor.

To conclude, our findings firstly indicate a potential impact of the physical office environment on the psychosocial work environment at the office workplace, previously not sufficiently studied. Secondly, they point at the importance of the office design’s ability to increase employee personal control—a key factor for employee well-being. The results are in an early stage in the study of associations between the physical and the psychosocial work environment. Nevertheless, the results may contribute to a greater interest in psychosocial aspects in the office design practice. Also, the recognition of psychosocial aspects in design may have implications for education in architecture, which means that it needs to be considered more systematically. Therefore, our results could have practical implications for the occupational health and organizational psychology practice that can improve employee performance and well-being, as long requested in this field [13]. After all, a physically and psychologically healthy workforce is the most important requirement for a viable organization [1] (p. 462). 

## Figures and Tables

**Table 1:** Socio-demographic characteristics of participants (age, educational level) in different office designs split by gender.

Office Types	Cell Office	Shared- Room	Small Open Plan Office	Med.-Sized Open Plan Office	Large Open Plan Office	Hot- Desking ^1^	Combi- Office	Summary of Per-centages	Sign. Diff. btw Office Designs
Total Sample (*n*) = 4352 Women (*n*) = 2380 Men (*n*) = 1972	*n*_1_ = 2084	*n*_2_ = 817	*n*_3_ = 470	*n*_4_ = 229	*n*_5_ = 350	*n*_6_ = 321	*n*_7_ = 81		
**Gender**									≤0001 (***)
Women	1089 (46)	459 (19)	279 (12)	128 (05)	172 (07)	205 (09)	48 (02)	~100.0	
Men	995(50)	358 (18)	191 (10)	101 (05)	178 (09)	116 (06)	33 (02)	~100.0	
**Age** ^a^									≤0001 (***)
Women	51.5	50	48	47.5	46	48.5	47		
Men	52.5	49	49	47.5	48	48.5	52		
**Education** ^b,c^									
Women									≤0001 (***)
(1) Low level	210 (42)	105 (21)	49(10)	36 (07)	34 (07)	60 (12)	11 (02)	~100.0	
(2) Middle high level	377 (45)	161 (19)	96(11)	40 (05)	68 (08)	78 (09)	22 (03)	~100.0	
(3) High level	502 (49)	193 (19)	134 (13)	52 (05)	70 (07)	67 (06)	15 (01)	~100.0	
Men									≤0001 (***)
(1) Low level	284 (54)	101 (19)	44 (08)	16 (03)	29 (05)	45 (09)	9 (02)	~100.0	
(2) Middle high level	357 (47)	132 (18)	84 (11)	42 (06)	76 (10)	51 (07)	10 (01)	~100.0	
(3) High level	354 (51)	125 (18)	63 (09)	43 (06)	73 (11)	20 (03)	14 (02)	~100.0	

*Note:* Figures in parentheses are percentages calculated row wise. ^1^ = hot-desking is a not an office type, but a subgroup of the office type flex-office. ^a^ = mean age of employees in office design. ^b^ = number of male and female employees is based on the age analyses. ^c^ Educational levels: low level = up to senior high, middle high level = senior high school education, high level = college/university education. The significance levels refer to total analyses of differences between office designs with regard to gender, age, and educational level *** = *p* ≤ 0.001.

**Table 2:** Psychosocial factors (psychological demands, social support). Mean values of scores with 95% confidence intervals.

Psych. Demands	Office Designs	Adj.	%	95% CI	Impact of		Df	F	*p*-Value	Skewness = 0.07
		(*n*)	Crude (Adjust.)		Office Design *					Kurtosis = 0.11
Male (*n* = 1948)	1—Cell office	(984)	12.90 (12.97)	12.74–13.05	Reference	Office design	6	2.08	0.053	
	2—Shared-room office	(353)	**12.73 (12.68)**	**12.47–12.99**	**0.046**	Age	1	**44.57**	**<0.0001**	
	3—Small open plan	(191)	13.11 (13.05)	12.79–13.43	NS	Education	1	**66.10**	**<0.001**	
	4—Medium-sized open plan	(100)	12.72 (12.57)	12.25–13.19	NS					
	5—Large open plan	(174)	13.38 (13.25)	13.04–13.72	NS					
	6—Hot-desking ^a^	(113)	13.34 (13.31)	12.89–13.79	NS					
	7—Combi-office	(33)	12.85 (12.88)	11.97–13.72	NS					
Female (*n* = 2336)	1—Cell office	(1066)	12.99 (12.99)	12.82–13.15	Reference	Office design	6	**6.13**	**<0.0001**	
	2—Shared-room office	(448)	**12.74 (12.76)**	**12.47–12.99**	<0.0001	Age	1	**8.81**	**0.003**	
	3—Small open plan	(278)	13.62 (13.58)	13.31–13.94	NS	Education	1	**22.13**	**<0.0001**	
	4—Medium-sized open plan	(125)	13.76 (13.75)	13.33–14.19	NS					
	5—Large open plan	(171)	13.33 (13.30)	12.96–13.71	NS					
	6—Hot-desking ^a^	(204)	13.39 (13.41)	13.01–13.76	NS					
	7—Combi-office	(45)	**14.24 (14.24)**	**13.33–15.16**	**0.019**					
**Social support**	Office designs	Adj.	%	95% CI	Impact of		Df	F	*p*-value	Skewness = −0.43
		(*n*)	Crude (Adjust.)		office design *					Kurtosis = 0.49
Male (*n* = 1871)	1—Cell office	(919)	19.51 (19.49)	19.32–19.69	Reference	Office design	6	**4.04**	**0.0005**	
	2—Shared-room office	(343)	19.02 (19.03)	18.72–19.32	NS	Age	1	1.04	0.309	
	3—Small open plan	(188)	**18.65 (18.66)**	**18.23–19.06**	**0.008**	Education	1	1.92	0.167	
	4—Medium-sized open plan	(99)	19.41 (19.45)	18.85–19.98	NS					
	5—Large open plan	(177)	19.06 (19.09)	18.63–19.48	NS					
	6—Hot-desking ^a^	(112)	**18.69 (18.67)**	**18.13–19.24**	**0.036**					
	7—Combi-office	(33)	20.00 (20.00)	19.05–20.95	NS					
Female (*n* = 2299)	1—Cell office	(1034)	19.09 (19.09)	18.90–19.27	Reference	Office design	6	0.34	0.541	
	2—Shared-room office	(446)	18.81 (18.81)	18.52–19.11	NS	Age	1	0.30	0.582	
	3—Small open plan	(274)	18.97 (18.97)	18.61–19.33	NS	Education	1	0.45	0.502	
	4—Medium-sized open plan	(128)	19.14 (19.13)	18.60–19.68	NS					
	5—Large open plan	(170)	19.16 (19.14)	18.73–19.59	NS					
	6—Hot-desking ^a^	(199)	18.76 (18.75)	18.32–19.21	NS					
	7—Combi-office	(48)	18.69 (18.67)	17.70–19.67	NS					

*Note:* Multivariate linear regression analysis. Crude (no adjustment) and adjusted model presented. Adjusted model in parentheses controlled for education and age. * *p*-value adjusted for education and age. ^a^ = hot-desking is a not an office type, but a subgroup to the office type flex-office. Reference category (cell office) is marked with underlined text, significances are marked in bold text.

**Table 3:** Psychosocial factors (decision authority, skill discretion). Mean values of scores with 95% confidence intervals.

Decision Authority	Office Design	Adj.	%	95% CI	Impact of		Df	F	*p*-Value	Skewness = −0.59
		(*n*)	Crude (Adjust.)		Office Designs *					Kurtosis = −0.06
Male (*n* = 1956)	1—Cell office	(989)	6.78 (6.75)	6.71–6.86	Reference	Office design	6	**10.80**	**<0.0001**	
	2—Shared-room office	(352)	**6.53 (6.55)**	**6.40–6.66**	**0.009**	Age	1	**31.62**	**<0.0001**	
	3—Small open plan	(188)	6.28 (6.30)	6.10–6.45	NS	Education	1	0.11	0.744	
	4- Medium-sized open plan	(101)	6.37 (6.42)	6.10–6.64	NS					
	5—Large open plan	(185)	6.38 (6.42)	6.18–6.57	NS					
	6—Hot-desking ^a^	(115)	**5.94 (5.98)**	**5.66–6.22**	**0.0003**					
	7—Combi-office	(32)	6.18 (6.16)	5.74–6.62	NS					
Female (*n* = 2371)	1—Cell office	(1085)	6.36 (6.32)	6.28–6.44	Reference	Office type	6	**9.12**	**<0.0001**	
	2—Shared-room office	(457)	**6.13 (6.13)**	**6.00–6.26**	**0.037**	Age	1	**18.15**	**<0.0001**	
	3—Small open plan	(278)	**6.25 (6.27)**	**6.08–6.42**	**0.0007**	Education	1	**51.9**	**<0.0001**	
	4—Medium-sized open plan	(128)	5.76 (5.82)	5.52–6.00	NS					
	5—Large open plan	(172)	6.00 (6.08)	5.78–6.22	NS					
	6—Hot-desking ^a^	(203)	**5.65 (5.69)**	**5.44–5.85**	**0.002**					
	7—Combi-office	(48)	**5.54 (5.60)**	**5.06–6.02**	**0.026**					
**Skill discretion**	Office design	Adj.	%	95% CI	Impact of		Df	F	*p*-value	Skewness = −0.55
		(*n*)	Crude (Adjust.)		office designs*					Kurtosis = 0.89
Male (*n* = 1947)	1—Cell office	(982)	13.17 (13.19)	13.08–13.26	Reference	Office design	6	1.02	0.408	
	2—Shared-room office	(350)	13.24 (13.22)	13.08–13.39	NS	Age	1	**20.02**	**0.001**	
	3—Small open plan	(191)	13.37 (13.35)	13.19–13.56	NS	Education	1	0.95	0.329	
	4—Medium-sized open plan	(99)	13.29 (13.25)	13.01–13.58	NS					
	5—Large open plan	(176)	13.10 (13.07)	12.92–13.29	NS					
	6—Hot-desking ^a^	(116)	13.36 (13.35)	13.12–13.61	NS					
	7—Combi-office	(33)	13.39 (13.41)	13.00–13.79	NS					

*Note:* Multivariate linear regression analysis. Crude (no adjustment) and adjusted model presented. Adjusted model in parentheses controlled for education and age. * *p*-value adjusted for education and age. ^a^ = hot-desking is a not an office type, but a subgroup to the office type flex-office. Reference category (cell office) is marked with underlined text, significances are marked in bold text.

**Table 4:** Emotional health (emotional exhaustion). Mean values of scores with 95% confidence intervals underlined text, significances are marked in bold text.

Emotional Exhaustion	Office Design	Adj.	%	95% CI	Impact of		Df	F	*p*-Value	Skewness = 1.12
		(*n*)	Crude (Adjust.)		Office Designs *					Kurtosis = 0.6
Male (*n* = 1949)	1—Cell office	(986)	9.96 (10.05)	9.65–10.27	Reference	Office type	6	**2.49**	**0.021**	
	2—Shared-room office	(352)	10.15 (10.08)	9.63–10.68	NS	Age	1	**16.29**	**<0.0001**	
	3—Small open plan	(190)	11.13 (11.05)	10.36–11.90	NS	Education	1	1.22	0.269	
	4—Medium-sized open plan	(99)	10.34 (10.17)	9.19–11.49	NS					
	5—Large open plan	(175)	10.43 (10.29)	9.70–11.17	NS					
	6—Hot-desking ^a^	(115)	**11.68 (11.61)**	**10.58–12.78**	**0.007**					
	7—Combi-office	(32)	9.59 (9.65)	7.22–11.97	NS					
Female (*n* = 2351)	1—Cell office	(1076)	11.47 (11.52)	11.11–11.83	Reference	Office type	6	**3.07**	**0.005**	
	2—Shared-room office	(452)	11.55 (11.56)	11.02–12.08	NS	Age	1	**14.67**	**0.0001**	
	3—Small open plan	(276)	**12.95 (12.85)**	**12.18–13.72**	**0.009**	Education	1	**9.32**	**0.002**	
	4—Medium-sized open plan	(125)	11.30 (11.23)	10.37–12.22	NS					
	5—Large open plan	(171)	**11.17 (11.03)**	**10.27–12.07**	**0.038**					
	6—Hot-desking ^a^	(205)	11.80 (11.80)	11.01–12.59	NS					
	7—Combi-office	(46)	13.52 (13.44)	11.43–15.61	0.046					

*Note*: Multivariate linear regression analysis. Crude (no adjustment) and adjusted model presented. Adjusted model in parentheses controlled for education and age. * *p*-value adjusted for education and age. ^a^ = hot-desking is a not an office type, but a subgroup to the office type flex-office. Reference category (cell office) is marked with bold.

**Table 5:** Office Designs—Including six office types and one sub-group to an office type.

Architectural Features	Functional Features
1. Cell office (individual office room)	
- The plan layout is characterized by corridors (single or double corridor system)	- Most equipment is in the own room. Work is concentrated and independent
- Individual room has access to a window	
2. Shared room office: (2–3 people share room)—It is sometimes a consequence of lack of workspace.	
- Workstations freely arranged in the room - For privacy reasons, sometimes screens or other divisional elements between workstations	Team-based work or people with similar work assignment work share room
- No individual window, shares with room mate(s)	- Most equipment outside of room, team-based shared rooms tend to have own equipment in room
*Traditional open-plan offices:* Employees share workspace in various configurations. Open-plan offices exist in three sub-categories:	
3. Small open plan office: (4–9 people share workspace) 4. Medium-sized open plan office (10–24 share workspace) 5. Large open plan office (>24 people share workspace)	
- Shared workspaces within the office	- Flexible for organizational changes
- Plan layout is open, based on an open flow of workspaces instead of corridor systems	- Routine-based work - Low level of interaction between employees
- Workstations freely arranged in the room or in rows in a larger workspace	- Often no amenities at workstation
*Activity-based and flexible office types:* ^a^	
6. Hot-desking office (sub-group to the office type Flex-office, holding only some of its features)	
- Plan layout is open	- Flexible for organizational changes - Dimensioned for <70% of the workforce - The choice of workstation is free, has the option to work - outside of office as well - Good information communication technology (ICT) is a - necessity as the common computer system is accessible - from all workstations within the office
	- Independent work, not always with high autonomy or decision authority
7. Combi-office: (team work and sharing of workspace and common facilities)	
- No strict spatial definition, personal workstations can be either individual rooms or open plan office - Back up spaces for work activities not suitable to carry out at the personal workstation - Extra focus on rooms for group activities such as: project rooms (to be booked for longer periods) team and meeting rooms	- >20% of the work in the office not at personal work station - Sharing of common amenities in common spaces - Work is both independent as well as interactive team work with colleagues in - Work is both independent and interactive team work - Team moves around in the office on an “as-needed basis” - to take advantage of the wide range of common facilities

^a^ = The activity-based category of office types holds flex-office and combi-office. In this table the category name is kept although flex-office is not represented in the study, but its sub-group hot-desking office. This sub-group does not hold any of the architectural features of flex-office that are activity-supportive or functional features of flex-office such as independent work with a high degree of autonomy and decision authority.

**Table 6:** Graphical overview of the differences * in psychosocial factors at work (incl. psych. demands, social support, decision authority, skill discretion) and emotional health outcome (emotional exhaustion) in different office designs split by gender.

Outcome	Cell- Office (Ref.)	Shared- Room	Small Open Plan Office	Med.-Sized Open Plan	Large Open Plan Office	Hot-Desking ^a^	Combi-Office	Impact of Office Design per se
	f = 1073–77 m = 962–90	f = 455 m = 357	f = 191 m = 276	f = 128 m = 99–101	f = 172 m = 178	f = 204–5 m = 116	f = 48 m = 33–35	
**Emotional health outcome:**								
**Emotional exhaustion**								
Men						■■		*
Women			♦♦		◊		♦	**
**Psychosocial factors at work:**								
**Psychological demands**								
Men		□						─
Women		◊◊◊					♦	***
**Social support**								
Men			■■			■		***
Women								─
**Decision authority**								
Men		■■				■■■		
Women		♦	♦♦♦			♦♦	♦	
**Skill discretion**								
Men								─
Women					♦♦♦		◊	***

*Notes*: Synthesis is based on *linear multivariate* analysis adjusted for age and education. Cell office was used as reference category. * = Differences in strength of significance, not in highest and lowest reported value. When no symbol is presented, there are no significant outcomes for the specific variable. Three markers = *p*-value < 0.001, two markers =* p*-value < 0.01, One marker = *p*-value < 0.05. ^a^ = hot-desking is a not an office type, but a subgroup to the office type flex-office. **□**, ■ (squares) = men, **◊**, ♦ (diamonds) = women, **□**, **◊** (white symbol) = significantly positive value, ■, ♦ (black symbol) = significantly negative value.

## Data Availability

The data presented in this study are available on request from the corresponding author.

## Appendix A

**Table A1:** Socio-demographic characteristics of participants in different office designs split by gender.

Office Designs	Cell- Office	Shared- Room	Small Open Open Plan	Med.-Sized Open Plan	Large Open Plan Office	Hot- Desking ^1^	Combi- Office	Sign. Diff. btw Office Designs
Total Sample (*n*) = 4352 Women (*n*) ^a^ = 2380, Men (*n*) ^a^ = 1972	*n*_1_ = 2084	*n*_2_ = 817	*n*_3_ = 470	*n*_4_ = 229	*n*_5_ = 350	*n*_6_ = 321	*n_7_ =* 81	
**Job rank (SEI)**								**≤0.001**
Women								
(5) Unskilled manual workers	15 (1.4)	17 (3.7)	8 (2.9)	4 (3.2)	-	32 (15.6)	28 (8)	
(4) Skilled manual workers	12 (1.1)	14 (3)	5 (1.8)	6 (4.7)	-	35 (17.1)	39 (11)	
(3) White collar worker—lower level	242 (22)	129 (28.1)	54 (19.5)	28 (22)	43 (25.3)	32 (15.6)	45 (12.7)	
(2) White collar worker—middle level	367 (33.8)	184 (40.1)	147 (53.1)	58 (45.7)	70 (41.2)	81 (39.5)	118 (3.3)	
(1) White collar worker—higher level	427 (39.4)	109 (23.7)	59 (21.3)	29 (23)	53 (32)	20 (9.6)	103 (29)	
(0) Self-employed	3 (0.3)	-	-	-	-	1 (0.5)	1 (2.8)	
Missing information	19 (1.8)	6 (1.3)	4 (1.4)	2 (1.6)	4 (2.6)	4 (1.95)	20 (5.65)	
Summary of percentage	~100.0	~100.0	~100.0	~100.0	~100.0	~100.0	~100.0	
Men								
(5) Unskilled manual workers	34 (3.4)	28 (7.9)	6 (3.1)	2 (2)	4 (2.3)	26 (22.4)	2 (6)	
(4) Skilled manual workers	43 (4.6)	39 (11)	10 (5.2)	5 (5)	5 (2.8)	25 (21.6)	4 (12)	
(3) White collar worker—lower level	137 (13.9)	45 (12.7)	23 (12)	7 (6.9)	9 (5.1)	15 (12.9)	1 (3)	
(2) White collar worker—middle level	338 (34.2)	118 (33)	97 (50.8)	47 (46.5)	69 (39)	24 (20.7)	10 (30)	
(1) White collar worker—higher level	399 (40.4)	103 (29.1)	51 (26.7)	36 (35.6)	83 (47)	20 (17.2)	16 (48.5)	
(0) Self-employed	13 (1.3)	1 (0.3)	-	-	-	2 (1.7)	-	
Missing information	24 (2.4)	20 (5.7)	4 (2.1)	4 (4.0)	7 (4)	4 (3.5)	-	
Summary of percentage	~100.0	~100.0	~100.0	~100.0	~100.0	~100.0	~100.0	
**Supervisory position**								**≤0.001**
Women								
(1) No supervisory position	697 (65)	308 (69)	210 (75.5)	98 (77)	113 (67)	136 (68)	21 (44.7)	
(2) Supervisory position not manager	140 (13)	84 (19)	46 (16.5)	19 (15)	33 (20)	50 (25)	21 (44.7)	
(3) Manager without staff responsibility	33 (3)	10 (2)	8 (3)	3 (2)	5 (3)	2 (1)	1 (2)	
(4) Manager with staff responsibility	202 (19)	42 (9.5)	14 (5)	8 (6)	17 (10)	13 (6)	4 (8.5)	
Summary of percentage	~100.0	~100.0	~100.0	~100.0	~100.0	~100.0	~100.0	
Men								
(1) No supervisory position	447 (46)	189 (54)	121 (64)	56 (57)	100 (57.5)	72 (63)	15 (45.5)	
(2) Supervisory position not manager	138 (14)	92(26)	31 (16)	23 (23)	39 (22.5)	28 (25)	8 (24)	
(3) Manager without staff responsibility	75 (7)	14 (4)	15 (8)	6 (6)	16 (9)	5 (4)	5 (15.2)	
(4) Manager with staff responsibility	321 (33)	57 (16)	23 (12)	14 (14)	19 (11)	9 (8)	5 (15.2)	
Summary of percentage	~100.0	~100.0	~100.0	~100.0	~100.0	~100.0	~100.0	

*Note:* Figures in parentheses are percentages calculated column wise. ^1^ = hot-desking is a not an office type, but a subgroup of the office type flex-office. ^a^ = number of male and female employees is based on the age analyses in Table 1.
